# Cryo-EM study of bacteriophage N4 virion RNA polymerase

**DOI:** 10.1128/jb.00208-26

**Published:** 2026-07-27

**Authors:** Manju Narwal, Yeonoh Shin, Katsuhiko S. Murakami

**Affiliations:** 1 Department of Biochemistry and Molecular Biology, Penn State University 189499, University Park, Pennsylvania, USA; 2 Huck Institutes of the Life Sciences, Center for Structural Biology, Penn State University 124474, University Park, Pennsylvania, USA; 3 Huck Institutes of the Life Sciences, Center for RNA Molecular Biology, Penn State University 124474, University Park, Pennsylvania, USA; Southern University of Science and Technology, Shenzhen, Guangdong, China

**Keywords:** bacteriophage N4, virion-encapsidated RNA polymerase, cryo-electron microscopy, single-particle reconstruction

## Abstract

Coliphage N4 employs a unique infection and transcription strategy in which early gene expression is driven by a virion-encapsidated RNA polymerase (vRNAP) that is injected into the host cytoplasm upon infection. Despite extensive biochemical and crystallographic studies of the polymerase domain of vRNAP, the structural organization and regulatory roles of the N-terminal domain (NTD) and C-terminal domain (CTD) regions of the 3,500-residue-long whole enzyme have remained unresolved. Here, we report the cryo-electron microscopy (cryo-EM) structures of full-length N4 vRNAP in its apo state and in a transcription initiation complex (TIC) with promoter DNA and initiating nucleotides. The apo structure reveals a modular architecture in which an α-helical CTD packs against the Pol domain to stabilize an autoinhibited conformation characterized by occlusion of the nucleotide-binding site through tight contact between the plug module and motif B loop. In contrast, promoter binding induces conformational rearrangements that displace the motif B loop from the active site and separate the CTD from the Pol domain. The NTD is unresolved in both states, consistent with substantial intrinsic flexibility, and supporting its proposed role in membrane association and genome injection. Structural modeling suggests that domain segmentation and conformational plasticity may enable translocation of vRNAP through the ~30 Å wide phage tail channel during infection. Together, these results define the molecular architecture of full-length vRNAP and establish a structural framework for understanding how the conformational transition of vRNAP is coupled to its ejection, DNA injection, and early gene expression.

**IMPORTANCE:**

This study investigates the structure of full-length bacteriophage N4 virion RNA polymerase (vRNAP), one of the largest known single-subunit RNA polymerases. The functions of its extensive N- and C-terminal regions remained unknown. Our work uncovers how the C-terminal domain regulates polymerase activity through a structural “switch” that locks the enzyme in an inactive state until it recognizes its promoter DNA. These findings explain how the phage prevents premature transcription and ensures precise control of early gene expression during infection. By integrating structures with the architecture of the N4 phage particle, we propose a mechanism by which this vRNAP is transported through the narrow phage tail into the host cell. Together, this work provides fundamental insight into phage transcription and viral gene regulation.

## INTRODUCTION

Bacteriophages employ diverse strategies to ensure rapid and ordered gene expression following genome delivery into host cells. *Escherichia coli* bacteriophage N4 and related members of the family *Schitoviridae* (1) are unique in that early transcription is independent of host RNA polymerase (RNAP). Instead, N4 injects a virion-encapsidated RNAP (vRNAP) into the host cytoplasm (2). This enzyme initiates transcription from three early promoters (P1, P2, and P3) located near the left end (~8 kb) of the 70.5-kb linear N4 genome, thereby triggering a transcriptional cascade that governs middle and late gene expression, genome replication, virion assembly (including vRNAP packaging), and host lysis.

N4 vRNAP is an exceptionally large single-subunit RNAP (~3,500 amino acids; ~382 kDa), among the largest members of the T7-like RNAP family. Although overall sequence similarity outside conserved catalytic motifs is limited, genetic and biochemical studies established its evolutionary relationship to T7 RNAP and mitochondrial RNAP (3). Unlike canonical T7-like enzymes, N4 vRNAP is inactive on double-stranded DNA and instead requires negative supercoil-induced extrusion of a promoter DNA hairpin (4, 5). Formation and stabilization of this hairpin depend on host DNA gyrase and *E. coli* single-stranded DNA-binding protein (EcoSSB) (6). Thus, N4 vRNAP represents a distinctive transcription system in which promoter recognition is governed primarily by DNA secondary structure rather than primary sequence.

**Fig 1:**
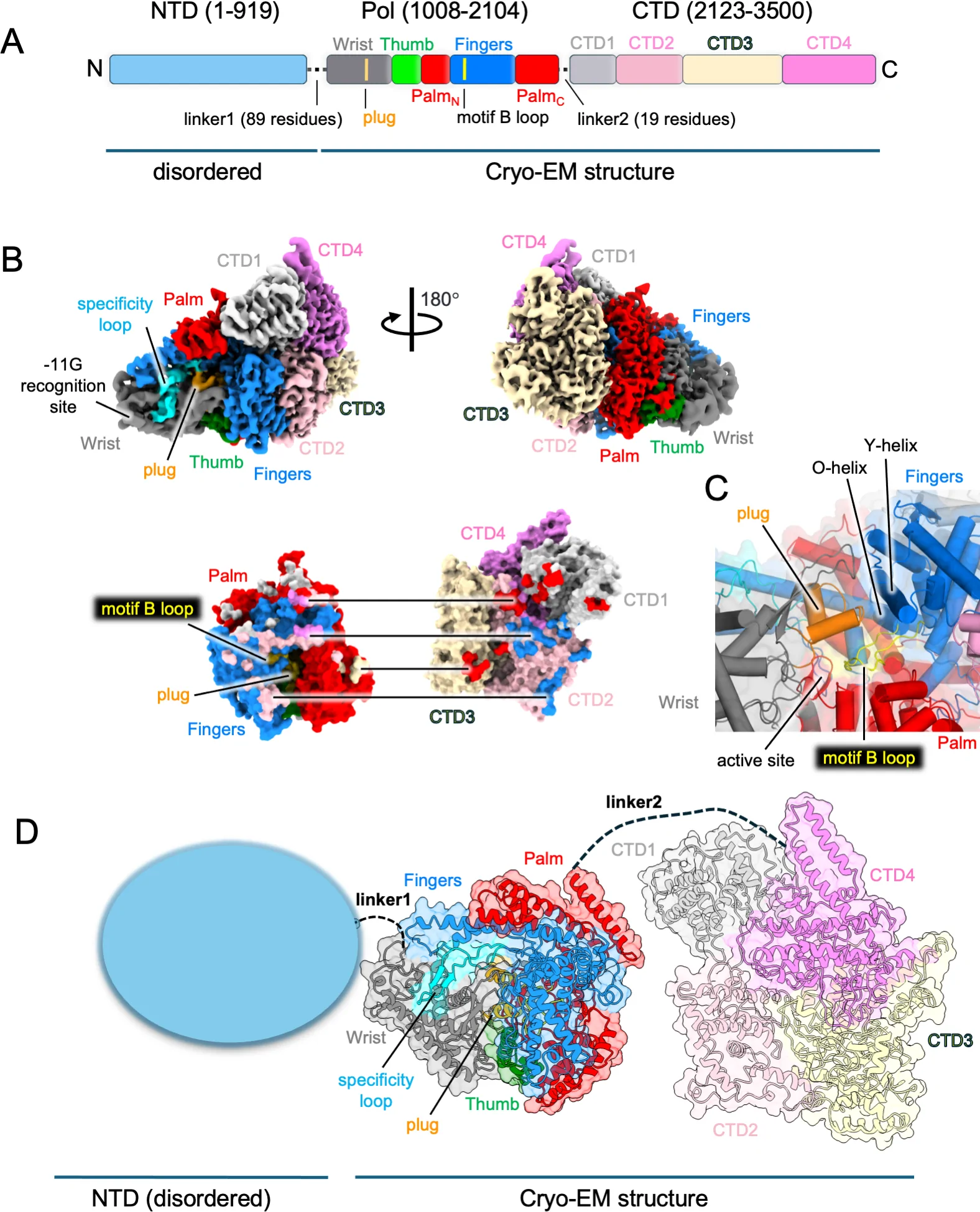
Cryo-EM structure and domain organization of the full-length bacteriophage N4 vRNAP. (**A**) Primary structure of the N4 vRNAP. The 3,500-residue unprocessed polyprotein vRNAP is organized into three major functional domains: the N-terminal domain (NTD; residues 1–919), the polymerase domain (Pol; residues 1,008–2,104), and the C-terminal domain (CTD; residues 2,123–3,500). The CTD is composed of four subdomains (CTD1–CTD4). The Pol domain includes the wrist, thumb, fingers, and palm subdomains. Key regulatory elements, including the plug, motif B loop, and specificity loop, are indicated. (**B**) (top) Cryo-EM density map of the full-length vRNAP. Two orthogonal views of the 3D reconstruction of vRNAP (right and left) are shown, revealing the compact packing of the CTD against the Pol domain. The cryo-EM map of NTD was not resolved. The structure highlights the positions of the specificity loop, the plug, and motif B loop modules that reside near the active site cleft. Rotations between views are indicated to illustrate the overall globular architecture of the complex. (bottom) Open-book view of the Pol and CTD interface. The Pol (left) and CTD (right) (surface representation) are separated and rotated by 180° relative to the bound complex to expose the interaction surfaces. Interface residues are colored in white (CTD1), pink (CTD2), and violet (CTD4) on the Pol domain, and in blue (fingers) and red (palm) on the CTD. (**C**) A magnified view of the plug and motif B interaction in the full-length vRNAP. Domains and motifs are indicated. (**D**) 3D structure of vRNAP showing NTD (cyan), Pol, and CTD (both cryo-EM structures). Three domains are linked by flexible linkers (linkers 1 and 2). The model is colored by domain as defined in (**A**).

Structural insight into N4 vRNAP has previously been limited to a proteolytically defined catalytic fragment (~1,100 amino acids) located in the central region of the full-length enzyme, also known as mini-vRNAP, but here referred to as the polymerase (Pol) domain (Fig. 1A) (3). X-ray crystal structures of apo and promoter-bound Pol domain revealed a canonical “cupped right-hand” architecture composed of wrist, thumb, palm, and fingers subdomains (7, 8). These studies also identified regulatory elements unique to N4 vRNAP, including the plug module and the motif B loop, which form tight interactions that exclude the template DNA-binding cleft in the apo state. The binding of the hairpin promoter induces large conformational rearrangements that reposition these elements, opening the cleft and converting the enzyme into a transcriptionally competent complex.

N4 vRNAP recognizes a hairpin-form DNA promoter by the Pol domain through multiple motifs that cooperatively engage both the sequence and shape of the promoter DNA. These include the −11 base recognition site and the β-intercalating hairpin at the N-terminal region, and the specificity loop on Fingers. Structural and biochemical studies showed that these elements establish extensive base-specific and phosphate backbone interactions with the hairpin promoter, thereby stabilizing a highly salt-resistant binary complex and enabling promoter-specific transcription initiation. In particular, the −11 guanine base (−11G) plays a critical role in promoter recognition and stable complex formation. Structural analyses showed that R119 forms base-specific interactions with the −11G base, while W129 stacks against the −11G base. This stacking interaction is especially important for the formation of the highly salt-resistant promoter-bound complex, highlighting the central role of the −11G position in hairpin promoter recognition by vRNAP (7, 8).

Despite detailed structural characterization of the Pol domain, the architecture and functional roles of the remaining ~2,400 amino acids—comprising the NTD and CTD—remain unresolved. Genetic and virological evidence suggests that the NTD participates in early genome injection and membrane association, whereas the CTD is required for encapsulation of vRNAP into progeny virions (2, 9). Early cryo-electron microscopy (cryo-EM) analyses of intact N4 particles localized vRNAP near the portal vertex (10), implying that the enzyme must traverse the narrow portal and tail channel during injection. More recent higher-resolution virion structures defined the dimensions of the portal complex and tail tube, but could not unambiguously resolve vRNAP (11), likely due to conformational heterogeneity. Thus, the structural organization of full-length vRNAP and the mechanism by which its auxiliary domains regulate transcription and virion-associated functions have remained undefined.

Here, we report near-atomic resolution cryo-EM structures of full-length N4 vRNAP in both apo and promoter DNA-bound states. The 3.4 Å apo structure enables accurate modeling of the Pol domain and a previously uncharacterized CTD, whereas the NTD (~1,000 residues) is not resolved, indicating substantial conformational flexibility. Structures of vRNAP bound to a P2 promoter hairpin DNA reveal conformational rearrangements associated with promoter engagement and CTD displacement. Together, these results define the modular architecture of full-length vRNAP and provide structural insight into how transcriptional regulation is integrated with virion biology.

## RESULTS

### Cryo-EM structure determination of full-length N4 vRNAP

To determine the structure of full-length N4 vRNAP, we expressed the recombinant enzyme in *E. coli* using an N-terminal His6-SUMO tag and purified it to homogeneity (Fig. S1A). The purified protein was subjected to single-particle cryo-EM analysis. Iterative two-dimensional and three-dimensional classifications yielded a well-defined reconstruction at an overall resolution of 3.4 Å (Fig. S2, Table 1).

**TABLE 1:** Cryo-EM data collection, refinement, and validation statistics for full-length N4 vRNAP

Cryo-EM data collection	Data collection parameters		
Magnification	81,000		
Voltage (kV)	300		
Electron dose (e^-^/Å^2^)	50		
Image pixel size (Å)	1.07		
Defocus range (μm)	−1.0 to −2.0		
Symmetry imposed	C1		
	apo-form		TIC
RNAP conformation	Closed plug	Open plug	
Number of micrographs	5,448		6,765
Final particle (no.)	51,340	57,669	327,675
PDB	11GP	11FW	11GO
EMBD	75681	75669	75680
3D structure refinement	apo-form		
Initial model used (PDB code)	2PO4		3Q22
Map resolution (Å) FSC threshold 0.143	3.43	3.35	2.82
Model composition			
Non-hydrogen atoms	19,069	19,057	8840
Protein residues	2453	2451	1090
Ligands	0	0	GTP:2; Mg:1
Bonds (RMSD)			
Length (Å) (# >4σ)	0.006	0.004	0.006
Angles (°) (# >4σ)	0.674	0.508	0.563
ADP (B-factors)			
Protein	180.02	165.29	159.46
Ligand	N/A^*a*^	N/A	210.47
Validation			
MolProbity score	1.99	1.71	1.25
Clash score	7.69	6.67	4.43
Rotamer outliers (%)	1.56	0.63	0.11
Ramachandran plot			
Favored (%)	93.54	95.05	97.89
Allowed (%)	6.34	4.95	2.11
Outliers (%)	0.12	0.00	0.00
Q-score	0.60	0.61	0.64
DAQ score (Cα only)			
Mean	0.762	0.774	0.774
Median	0.810	0.770	0.770
% <−0.5	1.02%	0.00%	0.00%
% <−1.0	0.29%	0.00%	0.00%

^*a*^ "N/A", not applicable.

The cryo-EM map displayed continuous density for the catalytic polymerase (Pol) domain and a large structured CTD (Fig. 1B). In contrast, no interpretable density was observed for approximately 1,000 N-terminal residues corresponding to the NTD. Mass spectrometry confirmed the presence of full-length vRNAP (Fig. S1B), indicating that the absence of density reflects intrinsic conformational flexibility or heterogeneity rather than proteolytic truncation.

### Overall architecture of full-length N4 vRNAP

The resolved structure reveals a modular organization in which the Pol domain (residues 1,008–2,104) is connected to the CTD (residues 2,123–3,500). The Pol domain adopts the canonical right-hand fold comprising wrist, thumb, palm, and fingers subdomains, consistent with previous X-ray structures of the isolated Pol domain (8). For clarity, the N-terminal subdomain within the Pol fold—previously referred to as NTD—is designated here as the “Wrist” subdomain to distinguish it from the NTD of full-length vRNAP (Fig. 1).

In the apo state of full-length vRNAP, the plug module and motif B loop form tight interactions that exclude the template DNA-binding cleft (Fig. 1C). This closed conformation prevents access by both template DNA and initiating nucleotides, indicating that the enzyme is catalytically inactive.

The CTD forms a compact, predominantly α-helical structure with approximate dimensions of 102 Å × 76 Å × 55 Å (Fig. 1B and D). It packs against the palm and fingers subdomains, covering one face of the Pol domain but leaving exposed the surface of vRNAP responsible for hairpin-form promoter DNA binding, including the −11G recognition site and the specificity loop. Surface area analysis by PDBePISA (12) indicates that only ~4.6% of the CTD solvent-accessible surface (2,641 out of 57,473 Å^2^) participates in interaction with the Pol domain (2,741 out of 45,429 Å^2^), suggesting that CTD association is likely reversible and dynamically regulated during infection and transcription.

The NTD, Pol domain, and CTD are connected by two flexible linkers (linker 1 and linker 2). The lack of well-resolved cryo-EM density for these linkers indicates substantial interdomain flexibility within the full-length vRNAP (Fig. 1D). Further domain analysis subdivides the protein into smaller structural modules (Pol: wrist and thumb/palm/fingers; CTD: CTD1, CTD2, CTD3, and CTD4), suggesting that vRNAP may adopt a segmented or modular configuration that facilitates its passage through the ~30 Å diameter size tail tube of the N4 phage during injection (11).

**Fig 2:**
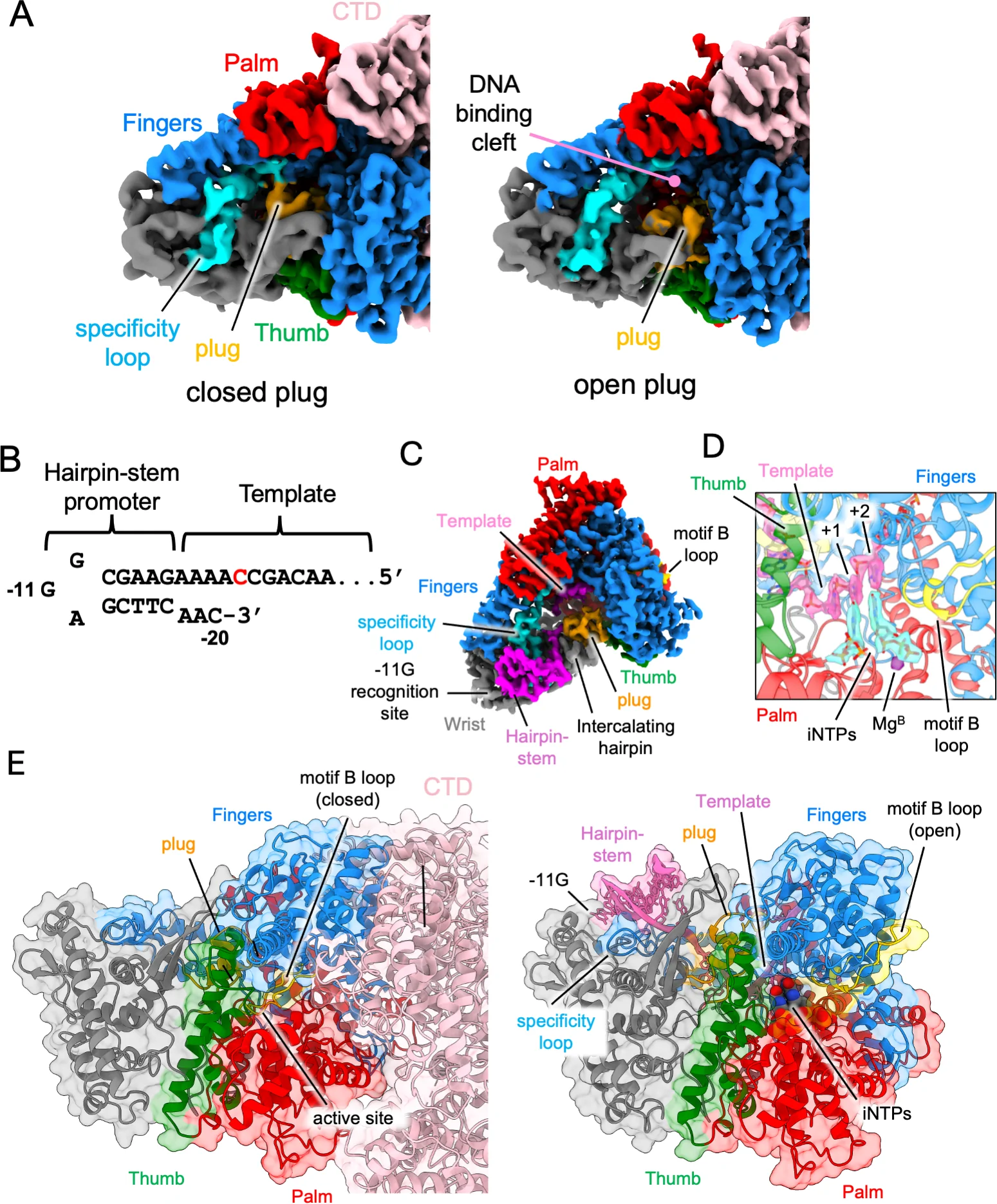
3D structures of apo-form and transcription initiation complex of vRNAP. (**A**) Comparison of two forms of the apo-form vRNAP in the “closed plug” and “open plug” states depicted as their cryo-EM density maps. Positions of the fingers, palm, thumb, CTD, specificity loop, and plug are indicated. (**B**) Schematic representation of the N4 early P2 promoter. The promoter consists of a conserved hairpin-stem including −11G followed by a single-stranded template region containing the transcription start site (+1, highlighted in red). (**C**) Cryo-EM density map of the vRNAP-promoter DNA-GTPs complex (TIC). The DNA hairpin-stem is recognized by the specificity loop and intercalating hairpin, while the template strand is directed toward the active site cleft. (**D**) A magnified view of the active site of TIC containing vRNAP, promoter DNA (hairpin-stem + template), incoming nucleotide (GTP), and Mg^2+^. Cryo-EM density map (transparent) of the template DNA (pink), iGTPs (cyan), and Mg^2+^ (magenta) is overlaid with the TIC structure (transparent cartoon model). Domains and motifs are indicated. (**E**) Conformational change of the motif B loop. Left: apo-form vRNAP structure (closed plug). The motif B loop (yellow) is in the “closed” conformation, and excludes a space of the active site for accommodating template DNA and nucleotide. Tight association of the CTD to the Pol domain prevents the motif B loop opening. Right: TIC structure. The motif B changes to the open conformation, making space for template DNA and GTP binding at the active site. Opening of the motif B loop requires the CTD separation from the Pol domain.

Three-dimensional classification of the apo-form vRNAP using a focused mask around the wrist revealed two conformational states distinguished by the position of the plug module (Fig. S2B). In the “closed plug” state, the plug resides within the DNA-binding cleft and forms tight contacts with the motif B loop, completely occluding the template DNA path. In the “open plug” state, the plug and intercalating hairpin shift toward the thumb subdomain, generating a cleft compatible with single-stranded template DNA binding (Fig. 2A). These two conformations are present at comparable levels in the final 3D reconstructions, with the closed plug and open plug states accounting for ~47.1% and ~52.9% of particles, respectively (Table 1). Opening the plug module likely represents the initial step in the formation of the transcription-ready promoter complex, as it presents the hairpin-form promoter DNA-binding site. Once the promoter DNA is bound and the template DNA gains access to the active site, a second conformational change might be triggered, facilitating the release of the CTD from the polymerase domain. Notably, the motif B loop remains in a closed conformation in both states, indicating that the vRNAP remains transcriptionally inactive even when it forms a binary complex with the hairpin-stem promoter.

### Structural transition of the N4 vRNAP during hairpin-stem promoter binding and transcription initiation

Our previous X-ray crystallographic analyses of the isolated Pol domain in its apo-form and in complex with promoter DNA revealed substantial conformational rearrangements upon promoter engagement, including an outward swing of the motif B loop during DNA binding (7, 8). To determine how these structural transitions are coordinated within the context of the full-length vRNAP and to elucidate the mechanism of hairpin-stem promoter recognition and transcription initiation, we assembled a complex containing full-length vRNAP, DNA containing P2 promoter (Fig. 2B), and initiating GTP. Cryo-EM reconstruction of this complex was determined at a nominal resolution of 2.8 Å (Fig. 2C; Fig. S3; Table 1). Although Mg²^+^ was included in the sample prior to grid preparation, the vRNAP accommodates two GTP molecules in the active site rather than forming a 2-mer RNA product, as observed in a previous X-ray crystallographic study of N4 mini-vRNAP (13). This structure represents the TIC. The cryo-EM density shows the nucleotide Mg²^+^ ion (Mg^B^, metal B), but lacks features corresponding to the catalytic Mg²^+^ ion (metal A, Mg^A^) (Fig. 2D), providing a structural explanation for the absence of RNA synthesis under these conditions.

In the TIC structure, the promoter hairpin is recognized by the specificity loop (Fingers) as well as the −11G recognition site and intercalating hairpin (wrist), and the template strand is directed toward the active site cleft (Fig. 2C) as observed in our previously published X-ray crystal structure of mini-vRNAP and promoter DNA complex (7). The motif B loop shifts from its inward, occluding position to an outward conformation, consistent with previous Pol-domain X-ray crystal structures (7). In this complex, both the NTD and CTD are disordered, indicating that promoter DNA binding and/or initiating nucleotide binding requires dissociation of the CTD from the Pol domain. Cryo-EM densities corresponding to the NTD and CTD could not be resolved in either 2D classification or after Gaussian (low path) filtering to the 3D reconstruction map, suggesting that both domains may become fragmented into smaller subdomains or adopt highly dynamic conformations upon promoter DNA binding. In the apo-form vRNAP structure containing CTD, the motif B loop occupies a space for the Pol active site, preventing its transcription reaction. Association of the CTD on the fingers/palm domains prevents the motif B from moving away from the active site (Fig. 2E left). In the TIC structure, the motif B loop swings outward, making a space for the template DNA and incoming nucleotides at the active site for transcription reaction (Fig. 2E, right). Separation of the CTD from the Pol domain requires avoiding its steric clash with motif B in its open conformation (Movie S1).

## DISCUSSION

The cryo-EM study presented here defines the molecular architecture and conformational landscape of the full-length N4 vRNAP and reveals how transcriptional regulation is embedded within its domain organization (Fig. 1). The X-ray crystallographic approach allowed us to investigate its 3D structure on only the compact and stable Pol domain of vRNAP that established the catalytic mechanism and hairpin-promoter recognition strategy of N4 vRNAP (7, 8, 14), but left unresolved how the large N-terminal and C-terminal regions contribute to injection, transcription regulation, and virion-encapsidation of vRNAP during N4 phage infection stages. Our cryo-EM analysis of the full-length vRNAP bridges this gap and demonstrates that domain architecture, conformational plasticity, and regulated CTD association to Pol domain and its separation to carry out early-stage transcription.

Full-length vRNAP exhibits a modular organization in which a catalytic Pol domain is flanked by a flexible NTD and a structured yet dynamically associated CTD (Fig. 2). The intrinsic disorder of the NTD observed in our cryo-EM structure reconstruction indicates that this region does not adopt a single stable conformation. Such flexibility is consistent with its proposed role in vRNAP and genome DNA injection and membrane-associated processes (2, 9, 15). In contrast, the CTD forms a compact α-helical structure that packs against the palm and fingers subdomains (Fig. 1B). Although the interface between CTD and Pol is limited, the interaction is sufficient to stabilize a catalytically inactive Pol conformation (Fig. 1C).

### Modular architecture of vRNAP, its ejection from phage virion into host cell, and transcription-coupled DNA injection

**Fig 3:**
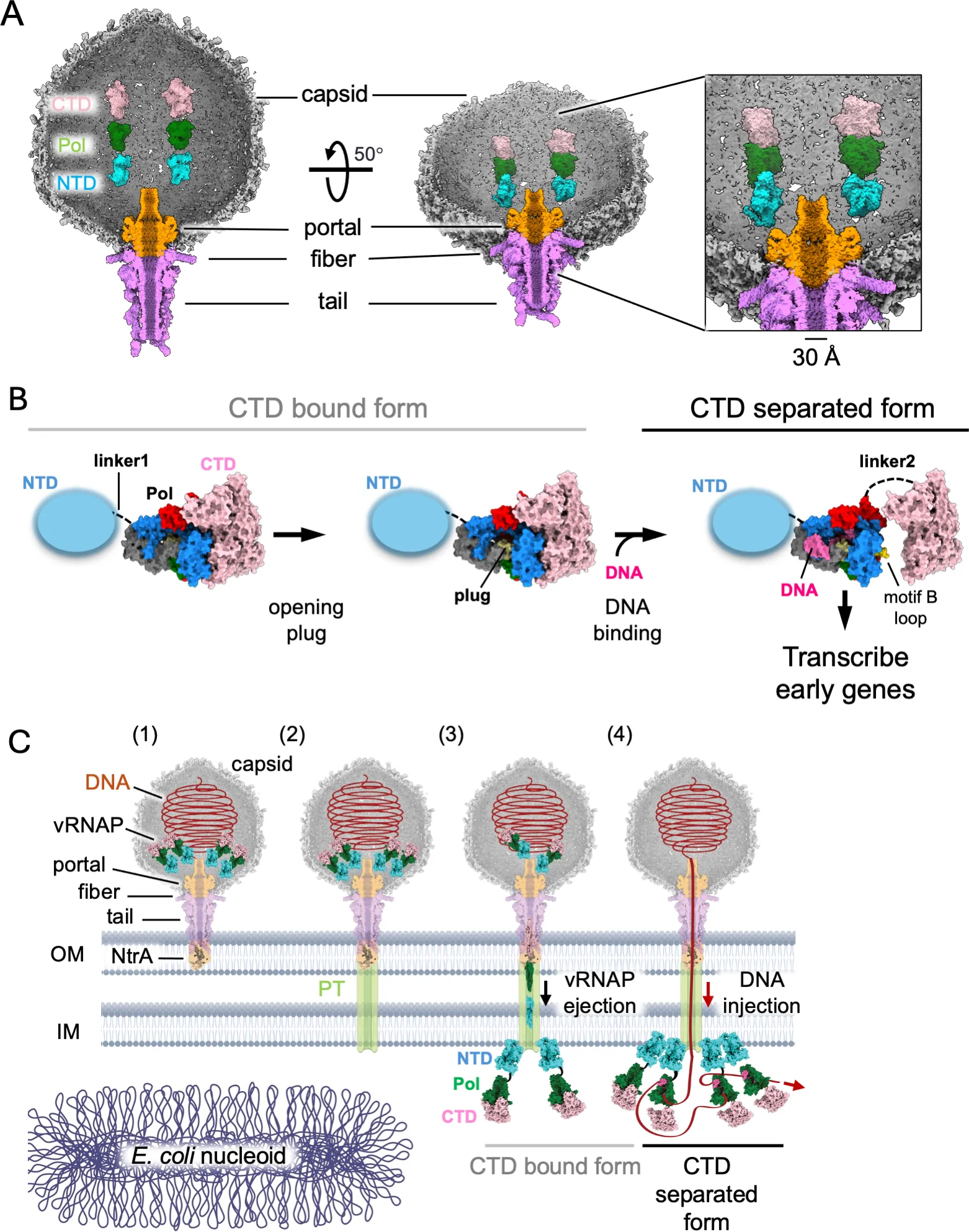
Model for N4 phage infection to *E. coli* and its vRNAPs and genome ejection. (**A**) Special arrangement of vRNAP within the N4 phage virion. Sliced view of the cryo-EM reconstruction of the bacteriophage N4 virion (11) is shown together with the 3D model of vRNAP, comprising NTD (cyan), Pol (green), and CTD (pink), localized near the portal complex. Key components of the viral particle, including the capsid, portal, tail, and fibers, are indicated. The view on the right represents a 50° rotation relative to the left view. This panel is a hybrid illustration generated by combining the N4 phage cryo-EM map with the vRNAP model, and both are displayed at the same scale. (**B**) Proposed stepwise model for the N4 early gene transcription. The process begins with the opening of the plug module of vRNAP, followed by DNA binding. Subsequent transition of the motif B loop separates the CTD from Pol to activate the enzyme for initiating early gene transcription. (**C**) Model for N4 phage infection and coordinated activation of vRNAP. (1) Receptor engagement. The N4 tail fiber binds the outer membrane receptor NfrA, triggering conformational rearrangements in the tail apparatus while the genome and vRNAP remain packaged within the capsid. (2) Formation of the trans-envelope complexes. Receptor binding promotes assembly of a periplasmic tunnel (PT) spanning the outer membrane (OM), peptidoglycan layer, and inner membrane (IM). This establishes a continuous channel connecting the virion interior to the host cytoplasm. (3) vRNAP ejection and membrane tethering. Prior to genome injection, vRNAPs are ejected through the PT into the cytoplasm in an inactive state. The NTD of vRNAP may associate with the inner membrane, spatially positioning the vRNAP beneath the injection channel while preventing nonspecific interaction with the host genome (*E. coli* nucleoid). (4) Phage DNA is subsequently injected into the cytoplasm. Arrival of the viral genome enables activation of membrane-tethered vRNAP, initiating transcription of early genes near the genome injection site, functioning vRNAP as a molecular motor to further inject the phage genome into the cytoplasm.

High-resolution cryo-EM structures of the N4 virion reveal that the portal and tail channel possess an internal diameter of approximately 30 Å (11). Comparison of this dimension with the sizes of individual vRNAP domains indicates that simple domain separation alone would be insufficient to permit passage through the tail tube (Fig. 3A). Instead, substantial conformational plasticity and/or further modular subdivision within each domain is likely required to enable vRNAP translocation through the portal–tail channel during injection.

Here, we propose a stepwise activation model for vRNAP during the early stage of infection (Fig. 3B and C; Movie S1). Following *E. coli* NfrA (N four resistance A)-mediated receptor engagement (16), N4 phage establishes a trans-periplasmic tunnel and outer membrane complex. vRNAPs are ejected into the cytoplasm in an inactive conformation and tethered via their NTD to the membrane-associated complex (9). Subsequent N4 genome injection occurs through the same tunnel, spatially coupling DNA entry to immediate activation of vRNAP and early transcription, including genes encoding the second N4 phage RNAP (N4 RNAPII) (1, 17). Since approximately four vRNAP molecules are encapsulated in the N4 phage virion (10), we propose that each vRNAP may recognize one of the four early promoters (P1–P4) located at the left end of the N4 genome. Transcription from these promoters facilitates the N4 genome injection by vRNAP as a molecular motor, functioning similarly to the ejection of the T7 phage genome by host *E. coli* RNAP transcription (18, 19).

## MATERIALS AND METHODS

### Cloning and plasmid construction of full-length N4 vRNAP

DNA encoding mini-vRNAP plus CTD cloned in pBAD vector was provided as a gift from Lucia Rothman-Denes and used as the template to construct a plasmid expressing the full-length N4 vRNAP. DNA encoding the NTD of vRNAP was chemically synthesized (GenScript) based on its amino acid sequence and codon-optimized for *E. coli* expression, with flanking regions to facilitate cloning. DNA fragments coding NTD, mini-vRNAP, and CTD fragment, and pSUMO expression vector were amplified using Q5 High-Fidelity DNA Polymerase (New England Biolabs). Fragments containing 20–30 bp overlapping regions were gel-purified and assembled by Gibson assembly at 50°C for 60 min to generate the full-length vRNAP construct in the pSUMO vector. The assembled plasmid was transformed into XL1-Blue-competent cells, and colonies were screened for the presence of the cloned insert. Positive clones were verified by sequencing.

### Expression and purification of the full-length N4 vRNAP

The pSUMO vector cloning full-length N4 vRNAP was transformed into *E. coli* LOBSTR-BL21(DE3)-RIL cells (Kerafast). A single colony was inoculated into 10 mL LB medium supplemented with kanamycin and grown overnight at 37 °C with shaking at 200 rpm. The culture was diluted 1:100 into fresh LB medium containing kanamycin and grown at 37°C to an OD_600_ of 0.6–0.8. Protein expression was induced with 0.5 mM IPTG, and cultures were incubated at 16°C for 16 h. Cells were harvested by centrifugation at 5,000 × *g* for 15 min at 4°C. Cell pellets were resuspended in buffer A containing 50 mM Tris-HCl (pH 8.0), 500 mM NaCl, and 1 mM PMSF and lysed by sonication on ice. The lysate was clarified by centrifugation at 20,000 × *g* for 30 min at 4°C. The supernatant was applied to a His-Trap column (Cytiva) pre-equilibrated in Buffer A, washed with buffer containing 50 mM imidazole, and eluted with buffer B (300 mM imidazole in 20 mM Tris-HCl [pH 8.0] and 200 mM NaCl).

Elution fractions containing full-length vRNAP were diluted with Buffer C (20 mM Tris-HCl, pH 8.0, 5% glycerol, 1 mM PMSF, 0.1 mM EDTA) to reduce the NaCl concentration to 50 mM. Ulp1 protease was added to remove the His-SUMO tag, and the cleaved sample was loaded onto the HiTrap Q column (Cytiva) for further purification. Fractions containing vRNAP were pooled and subjected to size-exclusion chromatography on a Superose 6 Increase column (Cytiva) equilibrated in gel filtration buffer (20 mM Tris-HCl [pH 8.0], 100 mM NaCl, and 2 mM MgCl_2_). Peak fractions corresponding to full-length vRNAP were collected and concentrated by Vivaspin concentrator (Saritorius). Protein concentration was determined spectrophotometrically at 280 nm, and protein was stored in −80°C freezer till further use

### LC-MS/MS Identification of the full-length N4 vRNAP

The ~380 kDa protein band corresponding to vRNAP was excised from a Coomassie-stained SDS–PAGE gel and submitted to the Penn State Huck Institutes of Life Science Proteomics and Mass Spectrometry Core Facility for identification of protein by LC-MS/MS. The gel slice was subjected to standard in-gel reduction, alkylation, and tryptic digestion, and the resulting peptides were analyzed by nanoLC coupled to high-resolution tandem mass spectrometry operating in data-dependent acquisition mode. MS/MS spectra were searched against the submitted vRNA polymerase sequence with carbamidomethylation (C) specified as a fixed modification and methionine oxidation as a variable modification. Peptide-spectrum matches were filtered at a false discovery rate (FDR) of ≤1%, and sequence coverage was determined by mapping identified peptides to the full-length protein.

### Cryo-electron microscopy grid preparation and data collection

Purified full-length vRNAP was concentrated to 1.5–2 mg/mL in gel filtration buffer. For TIC assembly, vRNAP was incubated with a synthetic single-stranded DNA oligonucleotide designed to form a hairpin P2 promoter structure. The reaction was performed in complex formation buffer (20 mM HEPES-NaOH, pH 7.5, 100 mM NaCl, 5 mM MgCl₂, and 1 mM DTT) and incubated at room temperature for 20 min to allow stable promoter binding prior to downstream analysis. The DNA hairpin promoter sequence used was 5′-CATTTAATTGAGAAGAACGAACAGC**C**AAAAGAAGCGGAGCTTCAAC-3′ (transcription start site is indicated as bold and underlined; DNA sequence forming hairpin-stem promoter is underlined). For cryo-EM sample preparation, 3 μL of protein sample was applied to glow-discharged Quantifoil R2/1 300-mesh copper grids (Electron Microscopy Sciences) and vitrified using a Vitrobot Mark IV (Thermo Fisher Scientific) at 4°C and 100% humidity. Grids were blotted for 3 s and plunge-frozen in liquid ethane cooled by liquid nitrogen before storage in liquid nitrogen until imaging.

Cryo-EM data were collected on a 300 keV Titan Krios transmission electron microscope (Thermo Fisher Scientific) equipped with a K3 direct electron detector (Gatan) operating in counting mode (National Cancer Institute’s National Cryo-EM Facility at the Frederick National Laboratory). Movies were recorded at a nominal magnification corresponding to a calibrated pixel size of ~0.8–1.0 Å/pixel. Data were collected over a defocus range of −1.0 to −2.5 μm. Each movie was dose-fractionated into 40 frames with a total accumulated dose of ~50 e⁻/Å².

Movies were motion-corrected and dose-weighted, and contrast transfer function (CTF) parameters were estimated using standard procedures in cryoSPARC (20). Cryo-EM workflows of the apo-form and TIC of vRNAP were described (Fig. S2 and S3). The best-resolved particle subset was subjected to non-uniform or homogeneous refinements and postprocessing by cryoSPARC to yield the final density map.

### Model building and refinement

Published apo-form mini-vRNAP model (PDB: 2PO4) (8) and TIC of mini-vRNAP (PDB: 3Q22) (13) were used as starting models. The initial model of the CTD of vRNAP was manually modeled by Coot (21). All structures were refined by Phenix with secondary structure restraint (22) and validated using MolProbity (23) and the DAQ program (24). Cryo-EM map display and figure preparation were performed using UCSF ChimeraX (25) and Pymol (Schrödinger).

## Data Availability

All data needed to evaluate the conclusions of this paper are present in the main text and/or the supplemental materials. The cryo-EM maps and the refined models were deposited in the Electron Microscopy DataBank (EMDB, https://www.ebi.ac.uk/emdb/) and the Protein Data Bank (PDB, https://www.rcsb.org/) (Table 1).

## SUPPLEMENTAL MATERIAL

Supplemental figures jb.00208-26-s0001.pdf Figures S1 to S3.

Movie S1 jb.00208-26-s0002.mp4 Proposed stepwise model for the N4 early gene transcription. The process begins with the opening of the plug module of vRNAP, followed by DNA binding. Subsequent transition of the motif B loop separates the CTD from Pol to activate the enzyme for initiating early genes transcription.

ASM does not own the copyrights to Supplemental Material that may be linked to, or accessed through, an article. The authors have granted ASM a non-exclusive, world-wide license to publish the Supplemental Material files. Please contact the corresponding author directly for reuse.
