# Supplementary material for: Cryo-EM study of bacteriophage N4 virion RNA polymerase
Source: J Bacteriol. 2026 Jul 27;208(8):e00208-26. doi: 10.1128/jb.00208-26 (PMC13492108; doi:10.1128/jb.00208-26)
Supplement: Supplementary file 1 [file jb.00208-26-s0001.pdf]

SI Fig. 1

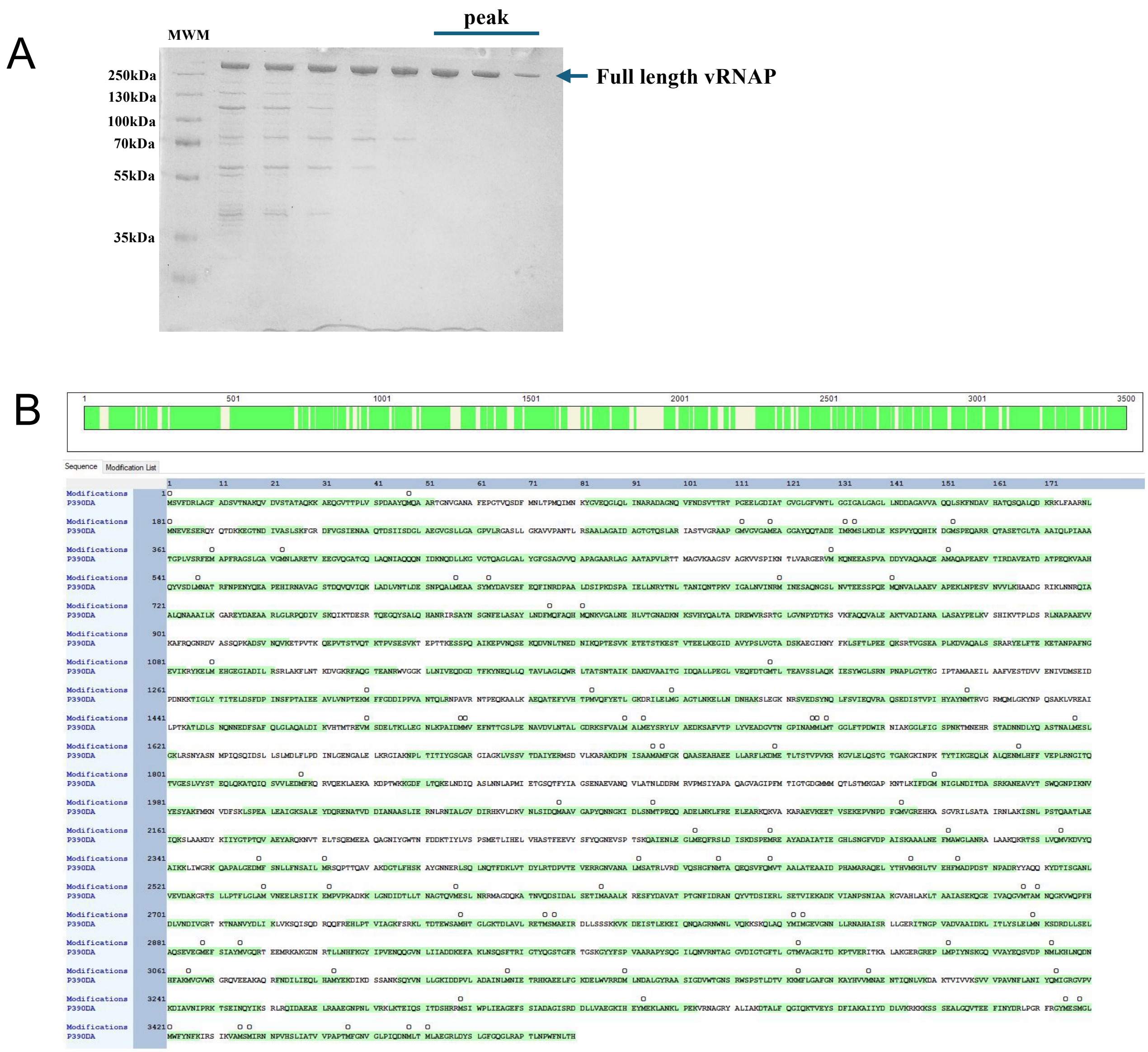

Supplementary Fig. 1: (A) Coomassie-stained SDS–PAGE gel of the gel-filtration column purified full-length vRNAP (3,500 aa, 382 kDa). Fractions used for cryo-EM grid preparation are indicated ("peak"). Molecular weight markers (kDa) are indicated. (B) LC-MS/MS sequence coverage of the purified full-length N4 vRNAP. The purified protein was analyzed by LC-MS/MS, with identified peptides highlighted in green across the 3500-residue sequence. Overall sequence coverage was 77.9%, with 231 unique peptides and 1203 peptide spectrum matches (PSMs). Peptides were detected (highlighted green) at all three domains (NTD, Pol and CTD) of the N4 vRNAP, confirming full-length protein was used to cryo-EM grid preparation.

SI Fig. 2

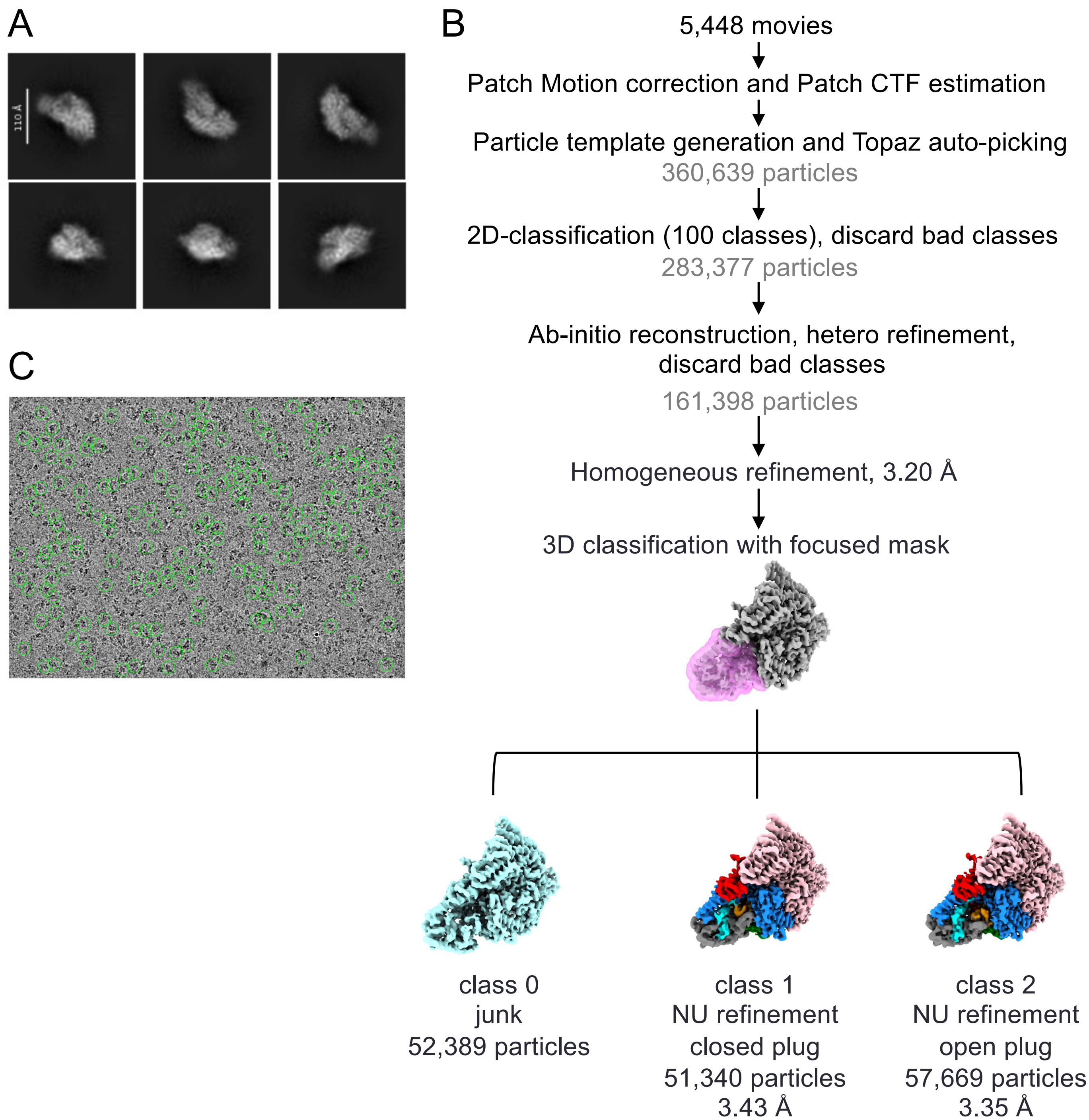

A
110 Å
B
5,448 movies
Patch Motion correction and Patch CTF estimation
Particle template generation and Topaz auto-picking
360,639 particles
2D-classification (100 classes), discard bad classes
283,377 particles
Ab-initio reconstruction, hetero refinement, discard bad classes
161,398 particles
Homogeneous refinement, 3.20 Å
3D classification with focused mask
class 0
junk
52,389 particles
class 1
NU refinement
closed plug
51,340 particles
3.43 Å
class 2
NU refinement
open plug
57,669 particles
3.35 Å
C

SI Fig. 2

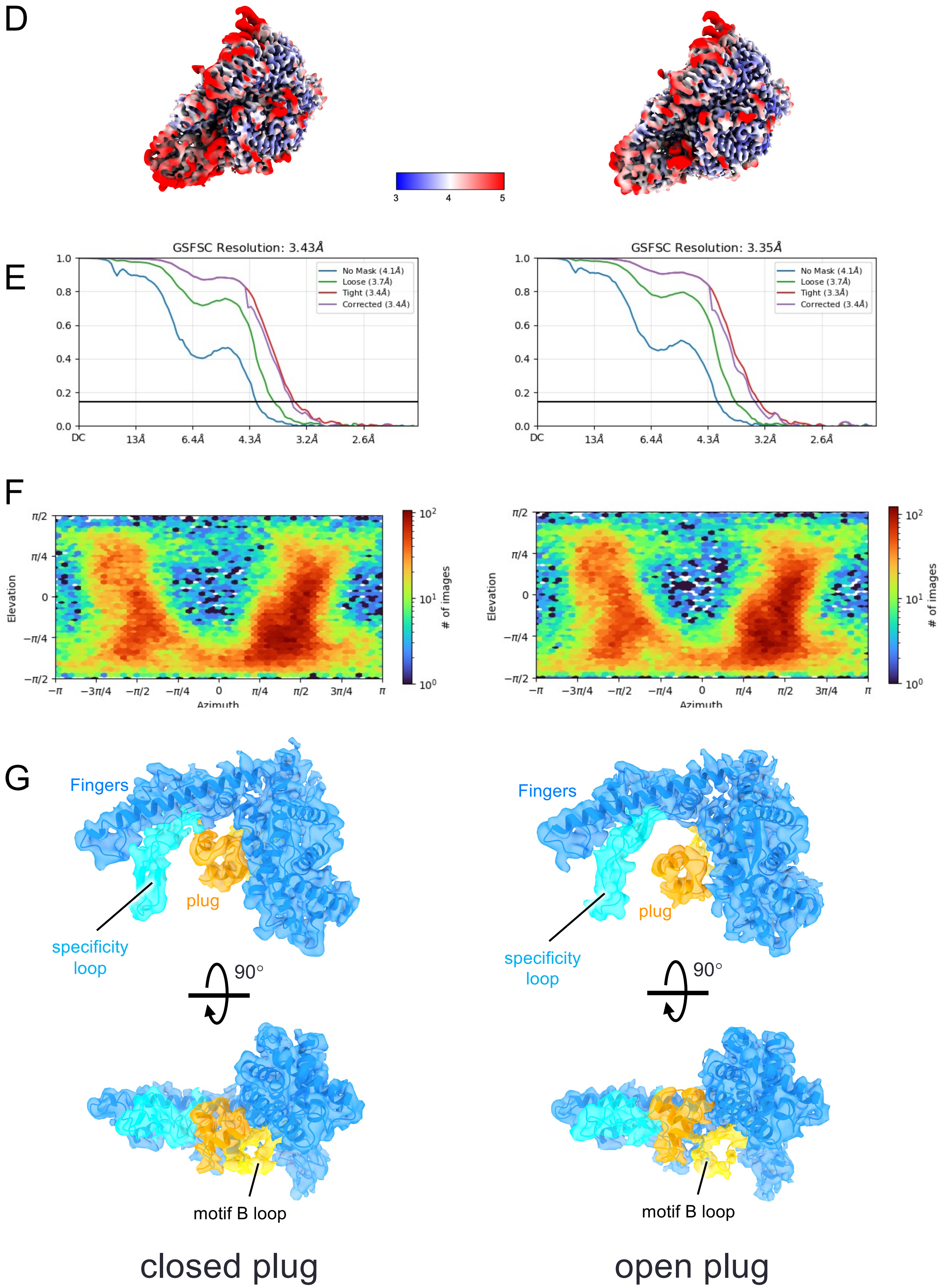

D
3
4
5
E
GSFSC Resolution: 3.43Å
No Mask (4.1Å)
Loose (3.7Å)
Tight (3.4Å)
Corrected (3.4Å)
GSFSC Resolution: 3.35Å
No Mask (4.1Å)
Loose (3.7Å)
Tight (3.3Å)
Corrected (3.4Å)
DC
13Å
6.4Å
4.3Å
3.2Å
2.6Å
F
Elevation
Azimuth
# of images
G
Fingers
plug
specificity loop
90°
motif B loop
closed plug
open plug

Supplementary Fig. 2: Cryo-EM workflow for the full-length N4 vRNAP data processing.

(**A**) Selected 2D classes. (**B**) Cryo-EM data processing and classification flow chart. Pink mesh depicts the mask around the wrist sub-domain during 3D classification. (**C**) Cryo-EM image of the full-length N4 vRNAP. Green circles indicate particles extracted from image for processing (200 Å diameter). (**D**) Local resolution maps. (**E**) Gold-standard FSC curves for the final 3D reconstruction. (**F**) The final Euler angle distribution. (**G**) Cryo-EM density map (transparent) with a final model (cartoon representation).

SI Fig. 3

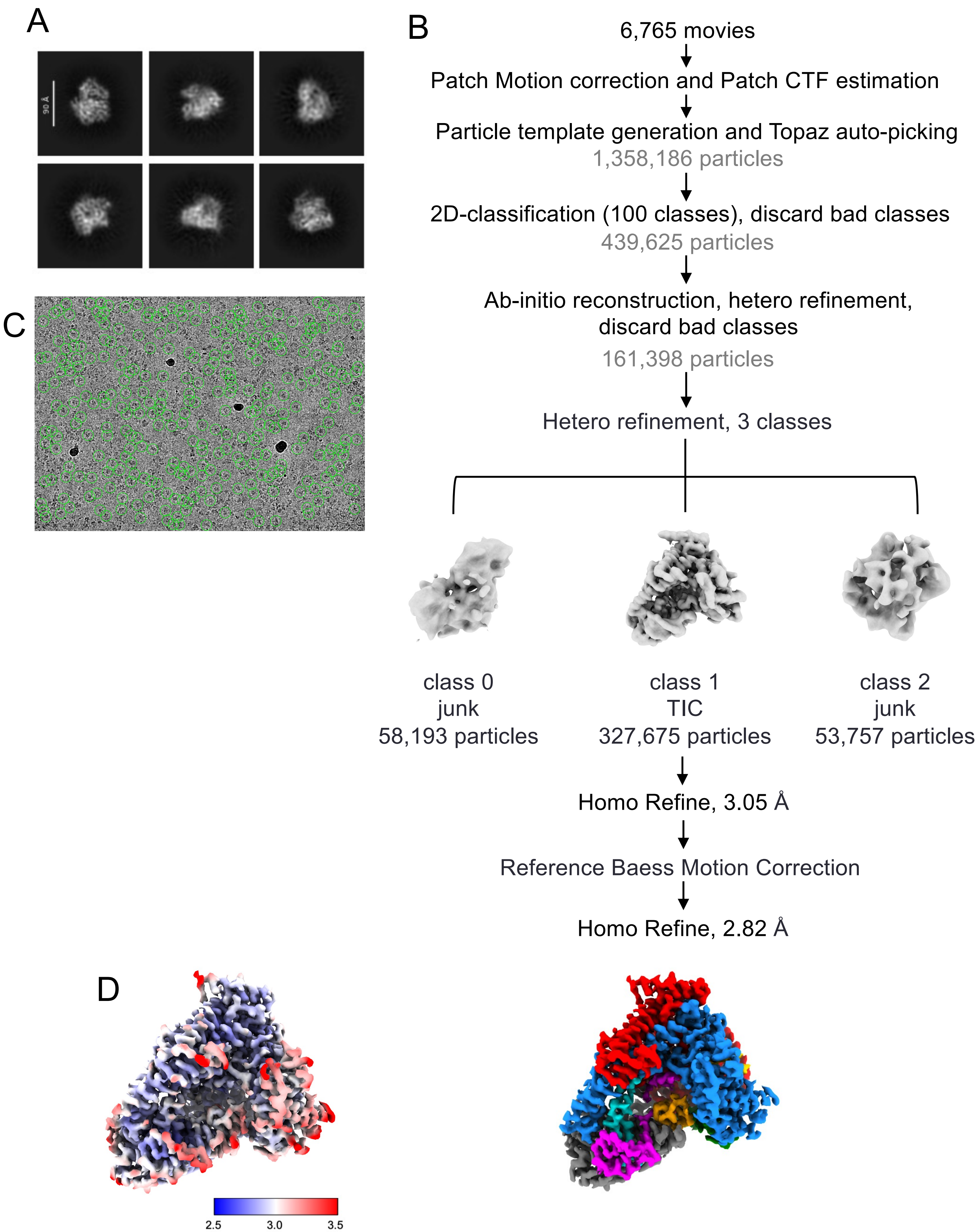

A
90 Å
B
6,765 movies
Patch Motion correction and Patch CTF estimation
Particle template generation and Topaz auto-picking
1,358,186 particles
2D-classification (100 classes), discard bad classes
439,625 particles
Ab-initio reconstruction, hetero refinement,
discard bad classes
161,398 particles
Hetero refinement, 3 classes
class 0
junk
58,193 particles
class 1
TIC
327,675 particles
class 2
junk
53,757 particles
Homo Refine, 3.05 Å
Reference Baess Motion Correction
Homo Refine, 2.82 Å
C
D
2.5
3.0
3.5

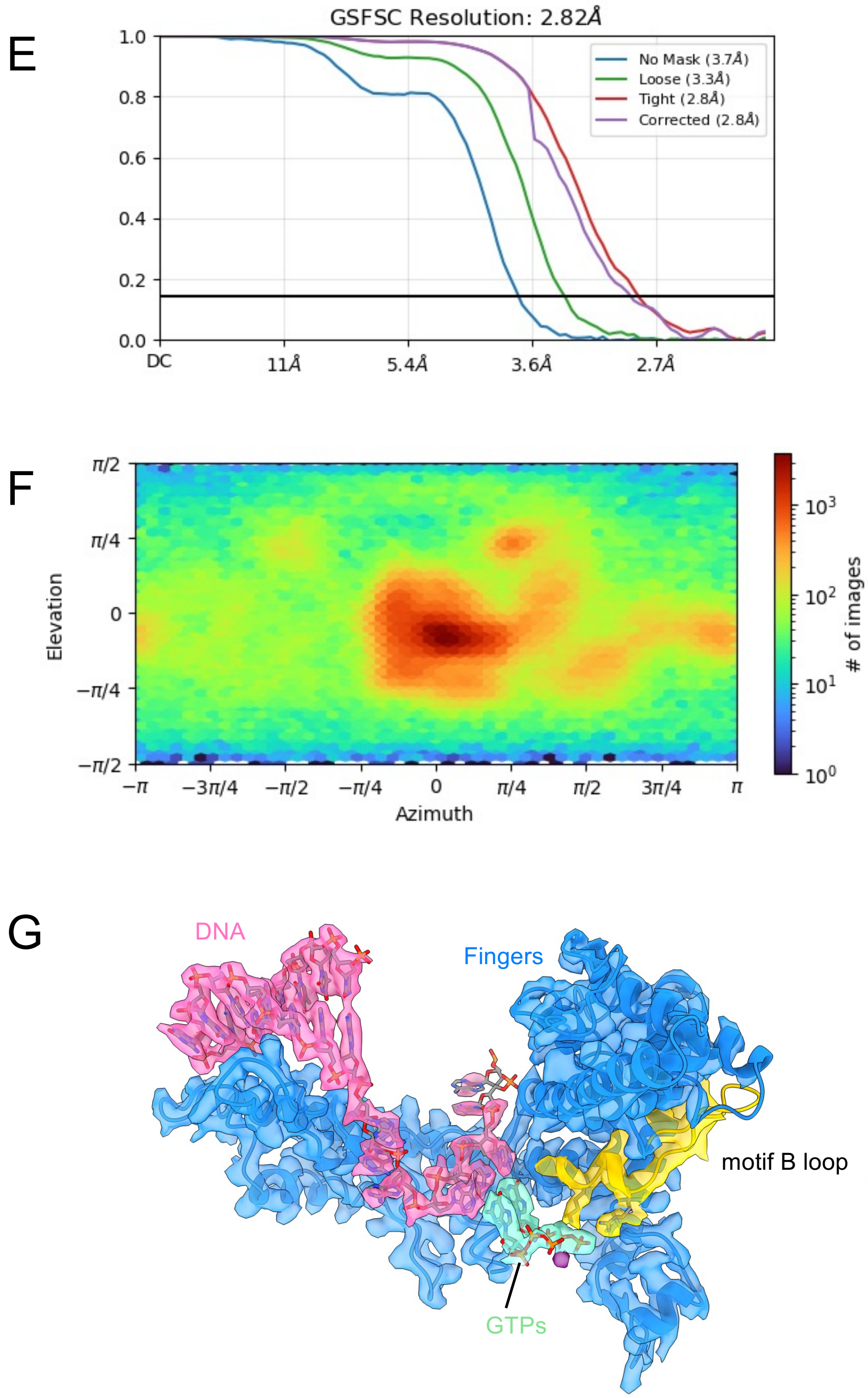

Supplementary Fig. 3: Cryo-EM workflow for the full-length N4 vRNAP, DNA and GTP complex (TIC) data processing. (**A**) Selected 2D classes. (**B**) Cryo-EM data processing and classification flow chart. (**C**) Cryo-EM image. Green circles indicate particles extracted from image for processing (200 Å diameter). (**D**) Local resolution maps. (**E**) Gold-standard FSC curves for the final 3D reconstruction. (**F**) The final Euler angle distribution. (**G**) Cryo-EM density map (transparent) with a final model (cartoon representation).

SUPPLEMENTARY MOVIE LEGEND

SMovie 1. Proposed stepwise model for the N4 early gene transcription. The process begins with the opening of the plug module of vRNAP, followed by DNA binding. Subsequent transition of the motif B loop separates the CTD from Pol to activate the enzyme for initiating early genes transcription.
